# Supplementary material for: Manipulating Weyl quasiparticles by orbital-selective photoexcitation in WTe2
Source: Nat Commun. 2021 Mar 25;12:1885. doi: 10.1038/s41467-021-22056-9 (PMC7994715; doi:10.1038/s41467-021-22056-9)
Supplement: Supplementary file 1 — Supplementary Information [file 41467_2021_22056_MOESM1_ESM.pdf]

Supplementary Information for

# Manipulating Weyl quasiparticles by orbital-selective photoexcitation in $WTe_2$

Meng-Xue Guan[1,2], En Wang[1,2], Pei-Wei You[1,2], Jia-Tao Sun[3, 1*]&Sheng Meng[1,2,4*]

[1]*Beijing National Laboratory for Condensed Matter Physics and Institute of Physics, Chinese Academy of Sciences, Beijing 100190, China.*

[2] *School of Physical Sciences, University of Chinese Academy of Sciences, Beijing 100190, China.*

[3] *School of Information and Electronics, Beijing Institute of Technology, Beijing 100081, China*

[4]*Songshan Lake Materials Laboratory, Dongguan, Guangdong 523808, China.*

These authors contributed equally: Mengxue Guan, En Wang.

*email: jtsun@iphy.ac.cn; smeng@iphy.ac.cn

**Supplementary Note 1: Applied laser waveform and the number of excited electrons**

The laser electric field $E(t)$ is described to be a Gaussian-envelope function,

$$E(t) = E_0\, cos(\omega t)\, exp\left[-\frac{(t-t_0)^2}{2\sigma^2}\right]. \quad (1)$$

Here, the width $\sigma$ is 10 fs, and photon energy $\omega$ is 0.6 eV. The laser field reaches the maximum strength $E_0 = 0.028$ V Å$^{-1}$ (2.8 MV/cm) at time $t_0 = 40$ fs (Supplementary Fig. 1a). Velocity gauge is used where the vector and scalar potential of the field $E(t)$ are $\mathbf{A}(t) = -c\int_0^t \mathbf{E}\,(t')dt'$ and $\Phi = 0$. Time evolution of the wavefunctions is then computed by propagating the Kohn-Sham equations in atomic units (a. u.),

$$i\frac{\partial}{\partial t}\psi_i(\mathbf{r},t) = \left[\frac{1}{2m}\left(\mathbf{p}-\frac{e}{c}\mathbf{A}\right)^2 + V(\mathbf{r},t)\right]\psi_i(\mathbf{r},t)\,. \quad (2)$$

Time-dependent current can be obtained as,

$$J(t) = \frac{1}{2i}\int_{\Omega} d\mathbf{r}\sum_i \{\psi_i{}^*(\mathbf{r},t)\nabla\psi_i(\mathbf{r},t) - \psi_i(\mathbf{r},t)\nabla\psi_i{}^*(\mathbf{r},t)\}. \quad (3)$$

As the ions are much heavier than electrons by at least three orders of magnitude, the nuclear positions are updated following the Newton's second law,

$$M_I\frac{d^2\boldsymbol{R}_I}{dt^2} = \sum_i \left\langle \psi_i \middle| \nabla_{\boldsymbol{R}_I}\left(\frac{1}{2m}\left(\boldsymbol{p}-\frac{e}{c}\boldsymbol{A}\right)^2 + V(\boldsymbol{r},t)\right) \middle| \psi_i \right\rangle, \quad (4)$$

where $M_I$ and $\mathbf{R}_\mathrm{I}$ are the mass and position of the $I^{\mathrm{th}}$ ion, respectively. Supplementary Eq.(2) and Eq. (4) represents the time-dependent coupled electron-ion motion. The time-dependent Kohn-Sham equations of electrons and the Newtonian motion of ions are solved simultaneously, with ionic forces along the classical trajectory evaluated through the Ehrenfest theorem.

The TDKS orbitals $\psi_{n,\mathbf{k}}(\mathbf{r},t)$ can be expressed by the combination of adiabatic basis $\{\varphi_{n,\mathbf{k}}(\mathbf{r},t)\}$

$$|\psi_{n,\mathbf{k}}(\mathbf{r},t)\rangle = \sum_{n'} c_{nn'\mathbf{k}}(t)|\varphi_{n',\mathbf{k}}(\mathbf{r},t)\rangle, \quad (5)$$

where $n$ and $n'$ denote the band index, $\mathbf{k}$ is the reciprocal momentum index, $c_{nn'\mathbf{k}}(t)$ is the time dependent coefficients. The adiabatic basis $\varphi_{n,\mathbf{k}}(\mathbf{r},t)$ are solved

by diagonalizing the Hamiltonian

$$H_{n,\mathbf{k}}(\mathbf{r},t)\left|\varphi_{n,\mathbf{k}}(\mathbf{r},t)\right\rangle = \varepsilon_{n,\mathbf{k}}(t)\left|\varphi_{n,\mathbf{k}}(\mathbf{r},t)\right\rangle \tag{6}$$

where $\varepsilon_{n,\mathbf{k}}$ is the eigenvalue.

During the lattice dynamics, based on the ground-state potential energy surface of a certain atomic configuration $\mathbf{R}(t)$, the adiabatic basis $\varphi_{n,\mathbf{k}}(\mathbf{r},t)$ is calculated on the fly at each ionic step. Therefore, the projection of the time-evolved wavefunctions ($|\psi_{n,\mathbf{k}}(t)\rangle$) on the basis of the adiabatic Kohn-Sham orbitals ($|\varphi_{n',\mathbf{k}}\rangle$) represent the state-to-state transition probabilities

$$P_{nn'\mathbf{k}}(t) = |c_{nn'\mathbf{k}}(t)|^2 = \left|\left\langle\varphi_{n',\mathbf{k}}(t)\middle|\psi_{n,\mathbf{k}}(t)\right\rangle\right|^2 \quad . \tag{7}$$

The population $q_n$ of band *n* is thus projected from TDKS orbitals as

$$q_n(t) = \frac{1}{N_{\mathbf{k}}}\sum_{\mathbf{k}}\sum_{n'\in n_{\mathbf{k},occ}} q_{n'\mathbf{k}}(t)P_{nn'\mathbf{k}}(t)\ , \tag{8}$$

where $n_{\mathbf{k},occ}$ is occupied state at $\mathbf{k}$ point and $N_{\mathbf{k}}$ is the total number of the **k**-points used to sample the Brillouin zone.

The dynamic of the excited electrons are calculated by

$$\Delta n_e(t) = N_e - \sum_{n}^{VB} q_n(t), \tag{9}$$

where all valence-band (VB) electrons are summed up and $N_e$ is the total number of electrons. The $\eta$ is used to denote the percentage of valence electrons that are pumped to specified unoccupied bands. For instance, $\eta = 1\%$ means $1\%$ of total valence electrons are pumped to conduction bands.

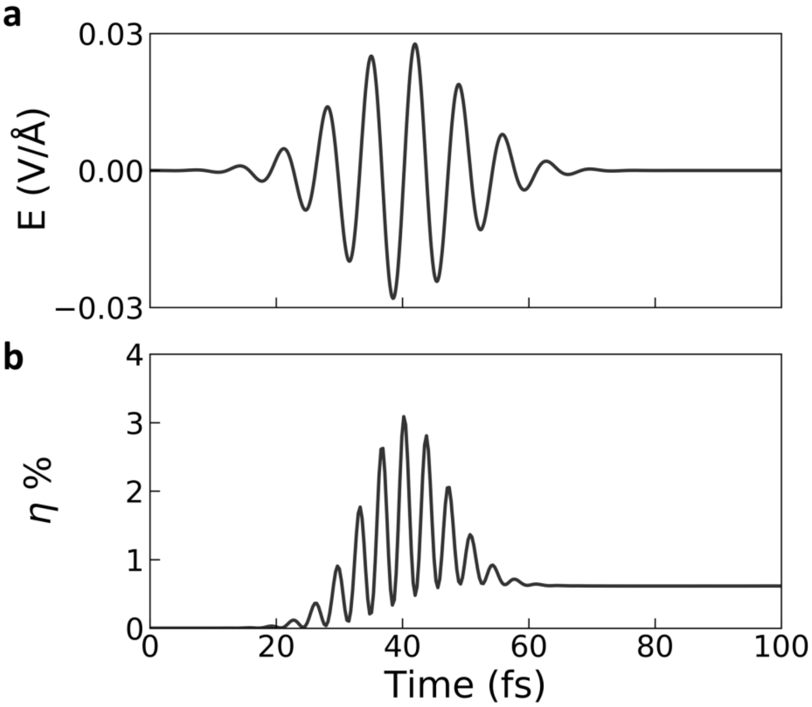


**Supplementary Fig. 1 | Laser waveform and the excited carriers. a,** Applied electric field along the in-plane direction of $T_d$-$WTe_2$ with the laser strength $E_0 = 0.028\,\text{V/Å}$. The pulse width is 10 fs and photon energy is 0.6 eV. **b,** The amount of excited electrons ($\eta$) upon photoexcitation from the valence bands to conduction bands with LP-a excitation. After the laser pulse ($t = 100$ fs), $\eta = 0.62\%$. For LP-b excitation, the fluctuation of excited electrons follows similar tendency as what we show here, but with $\eta = 0.18\%$.

**Supplementary Note 2: Optical transition selection rules**

The optical transition probability rate for an electron that is excited by a photon from the valence band to the conduction band can be described by the Fermi golden rule. Based on time-dependent perturbation theory, the transition probability from an initial state $|\psi_i\rangle$ to a final state $|\psi_f\rangle$ is

$$\Gamma_{if} = \frac{2\pi}{\hbar}\left|\langle\psi_f|H'|\psi_i\rangle\right|^2 \delta(E_f - E_i - \hbar\omega). \quad (10)$$

The operator for the interaction between the system and the electric field is

$$H' \approx \frac{eA}{m}\boldsymbol{\epsilon}\cdot\mathbf{p}, \quad (11)$$

where $\boldsymbol{\epsilon}$ is the laser polarization vector and $\mathbf{p} = \frac{\hbar}{i}\frac{\partial}{\partial\mathbf{r}}$ is the momentum operator. For the wavefunctions as Bloch waves, we have $\psi_i(\mathbf{r},t) = \mu_i(\mathbf{r},t)e^{i\mathbf{k}\cdot\mathbf{r}}$, and hence, $\Gamma_{if} \propto \left|\left\langle\psi_f\middle|\boldsymbol{\epsilon}\cdot\frac{\partial}{\partial\mathbf{r}}\middle|\psi_i\right\rangle\right|^2$. Therefore, the optical transition probability strongly depends on the details of the initial and final state wave functions and the polarization direction of the incident laser pulses. In practice, it is sufficient to analyze the symmetry of the transition dipole moment $M_{if} = \langle\psi_f|\boldsymbol{\epsilon}\cdot\mathbf{r}|\psi_i\rangle$ to determine a selection rule. If the symmetry of $M_{if}$ spans the totally symmetric representation of the point group to which the crystal belongs (i.e., an even function), then its integral over all space is not zero and the transition is allowed. Otherwise, the transition is forbidden.

The symmetry of the transition moment function is the direct product of the parity of its three components $\Gamma_f\otimes\Gamma_S\otimes\Gamma_i$. The symmetry characteristics of each component can be obtained from standard character tables. For $T_d$-$WTe_2$, the character table and the multiplication table are shown as Supplementary Table 1 and 2, respectively. Let us consider transition from a $p_x$ orbital of the Te atom to a $d_{z^2}$ orbital of the W atom, which have the symmetry of the $B_1$ and $A_1$ irreducible representation, respectively. For a laser pulse with polarization direction along the crystallographic *a*-axis (real-space *x*-axis), its irreducible representation is $B_1$. The direct product of the parities of these three components yields an $A_1$ irreducible symmetry representation (i.e., none of the symmetry operations changes it), therefore, the transition is allowed.

However, a laser pulse polarized along the crystallographic *b*-axis (real-space *y*-axis) leads to a symmetry characteristic of $A_2$, which is anti-symmetric under $\sigma_v(xz)$ and $\sigma_v'(yz)$ operations, and therefore, the integral of $M_{ij}$ over all space will be zero and the transition is forbidden. Some possible transition pathways near the Weyl nodes of $T_d$-$WTe_2$ are summarized in Supplementary Table 3.

**Supplementary Table 1**| The character table for the $C_{2v}$ symmetry point group

| | E | $C_2(z)$ | $\sigma_v(xz)$ | $\sigma_v'(yz)$ | linear, rotations | quadratic |
|---|---|---|---|---|---|---|
| $A_1$ | 1 | 1 | 1 | 1 | $z$ | $x^2, y^2, z^2$ |
| $A_2$ | 1 | 1 | -1 | -1 | $R_z$ | $xy$ |
| $B_1$ | 1 | -1 | 1 | -1 | $x, R_y$ | $xz$ |
| $B_2$ | 1 | -1 | -1 | 1 | $y, R_x$ | $yz$ |

**Supplementary Table 2**| The multiplication table for the $C_{2v}$ symmetry point group

| | $A_1$ | $A_2$ | $B_1$ | $B_2$ |
|---|---|---|---|---|
| $A_1$ | $A_1$ | $A_2$ | $B_1$ | $B_2$ |
| $A_2$ | $A_2$ | $A_1$ | $B_2$ | $B_1$ |
| $B_1$ | $B_1$ | $B_2$ | $A_1$ | $A_2$ |
| $B_2$ | $B_2$ | $B_1$ | $A_2$ | $A_1$ |

**Supplementary Table 3**| Optical transition selective rules in $T_d$-$WTe_2$

| $\Gamma_f$ | $\Gamma_S$ | $\Gamma_i$ | $\Gamma_f \otimes \Gamma_S \otimes \Gamma_i$ | Transition allowed? |
|---|---|---|---|---|
| $d_{z^2}$ | $x$ | $p_x$ | $A_1 \otimes B_1 \otimes B_1 = A_1$ | Yes |
| $d_{z^2}$ | $x$ | $d_{xz}$ | $A_1 \otimes B_1 \otimes B_1 = A_1$ | Yes |
| $d_{yz}$ | $y$ | $d_{z^2}$ | $B_2 \otimes B_2 \otimes A_1 = A_1$ | Yes |
| $d_{z^2}$ | $y$ | $p_x$ | $A_1 \otimes B_2 \otimes B_1 = A_2$ | No |
| $d_{z^2}$ | $y$ | $d_{xz}$ | $A_1 \otimes B_2 \otimes B_1 = A_2$ | No |
| $d_{yz}$ | $x$ | $d_{z^2}$ | $B_2 \otimes B_1 \otimes A_1 = A_2$ | No |

**Supplementary Note 3: Coherent phonon modes participating in the electron-phonon interactions upon photoexcitation**

The evolution in the density of photoexcited carriers on the highest valence band and the corresponding Fourier amplitude spectrum are shown within the timescale of picosecond (Supplementary Fig. 2a and 2b). It is clear that optical phonon modes in the frequency range of 2~6 THz play the most important role in the electron-phonon interactions, with the main peak located at 4 THz. Supplementary Fig. 2c shows the phonon dispersion of $T_d$-$WTe_2$ and the vibration patterns for some representative modes. For higher-frequency modes (~4 THz), the inter- or intralayer atoms have opposite movement tendency along the *b*-axis, which might couple with the interlayer shear mode (~0.23 THz), inducing the phase transition between the $T_d$ and the 1T′ or 1T′(*) phases.

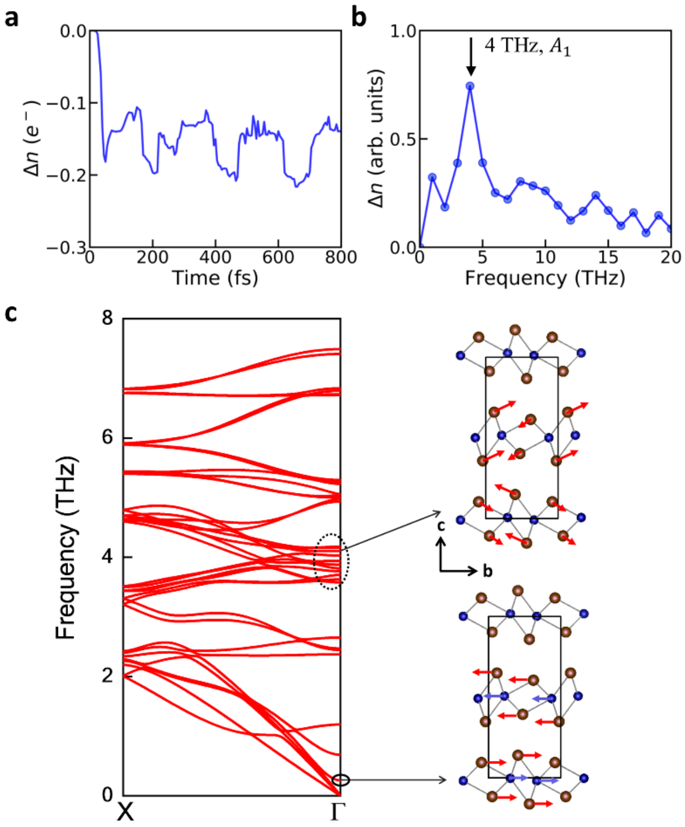


**Supplementary Fig. 2| Coherent phonon modes that participate in the phase transition. a,b,** Time evolution of carrier density on the highest valence band and the corresponding Fourier amplitude spectrum. **c,** Phonon dispersion of $T_d$-$WTe_2$ and the vibration patterns for some representative modes.

**Supplementary Note 4: Laser fluence dependence**

To demonstrate the fluence dependence, three laser pulses polarized along the crystallographic *b*-axis are applied to $T_d$-$WTe_2$ with different amplitudes. The corresponding interlayer shear displacements are shown in Supplementary Fig. 3. It is obvious that with the increase of laser amplitude, the atomic movements are accelerated and linearly dependent on the laser fluence.

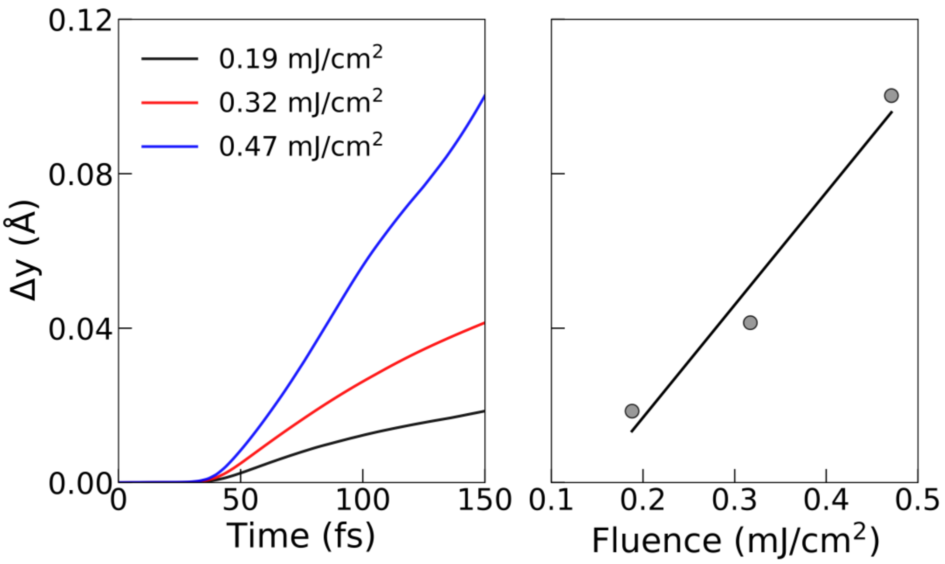


**Supplementary Fig. 3| Averaged displacement of the top layer atoms under various field fluences.** The laser duration are same as those shown in Supplementary Fig. 1a for all the laser pulses.

**Supplementary Note 5: Suitable photon energy that can induce polarization-anisotropic shear mode**

Several photon energies are tested to confirm that the polarization-anisotropic response is determined by the elaborate electronic excitations. We found that when the photon energy is in the range of 0.5~0.8 eV, similar selective excitation of WSMs will emerge, leading to the polarization dependent interlayer displacement. Supplementary Fig. 4 shows the results with three photon energies. Note that under smaller photon energy, (e.g., 0.5 eV), the interlayer displacement is larger, which might be ascribed to the fact that the transitions are closer to the Weyl nodes.

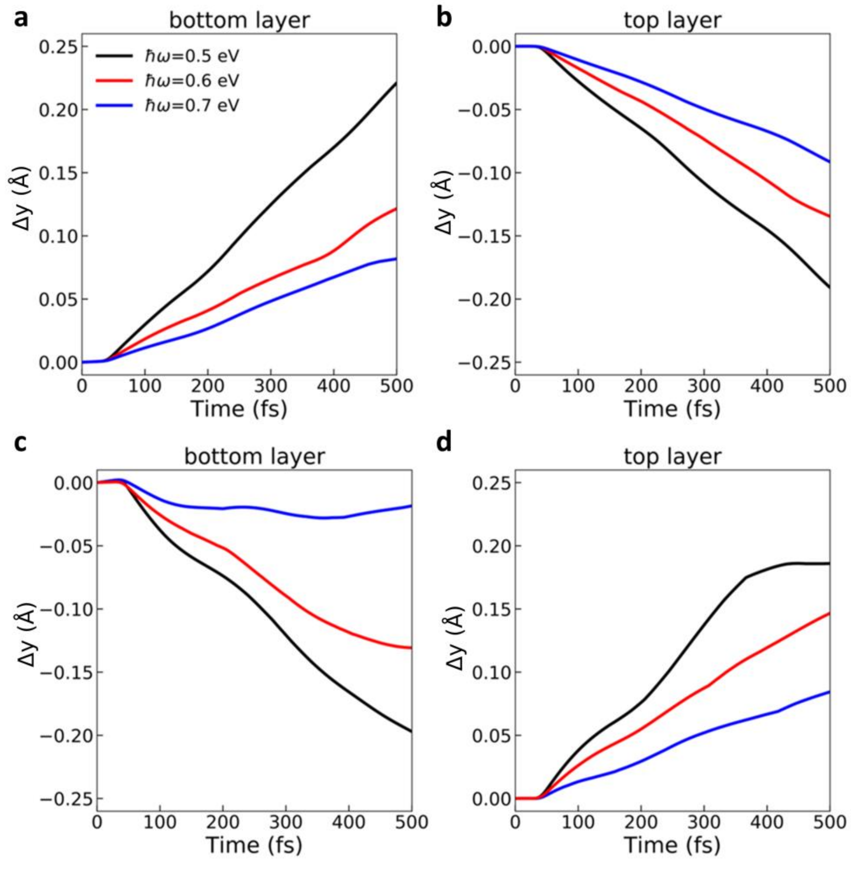


**Supplementary Fig. 4| Photon-energy dependence of interlayer shear displacement. a**,**b**, Time-evolutions of interlayer shear displacement of the bottom (**a**) and the top (**b**) layer under LP-a excitation. **c**,**d,** are analogous to **a,b**, but under LP-b excitation. The laser duration and intensity are same as those shown in Supplementary Fig. 1a for all the light pulses.

**Supplementary Note 6: Interlayer motion along *a*-axis and *c*-axis with the photon energy of 0.6 eV**

The interlayer displacements along *a*-axis and *c*-axis are monitored as well and can be described as

$$\Delta x(\mathrm{t}) = \frac{1}{N}\sum_{i=1}^{N}\{x_i(t) - x_i(0)\}, \tag{12}$$

$$\Delta z(\mathrm{t}) = \frac{1}{N}\sum_{i=1}^{N}\{z_i(t) - z_i(0)\}, \tag{13}$$

where $x_i(t)$ and $z_i(t)$ are the time-dependent positions of atom $i$ along the *a*-axis and *c*-axis, respectively. Comparing with the obviously movement along *b*-axis (Fig. 5a, b in the main text), negligible displacements are observed along these two directions (Supplementary Fig. 5), indicating that the shear motion is only along the *b*-axis.

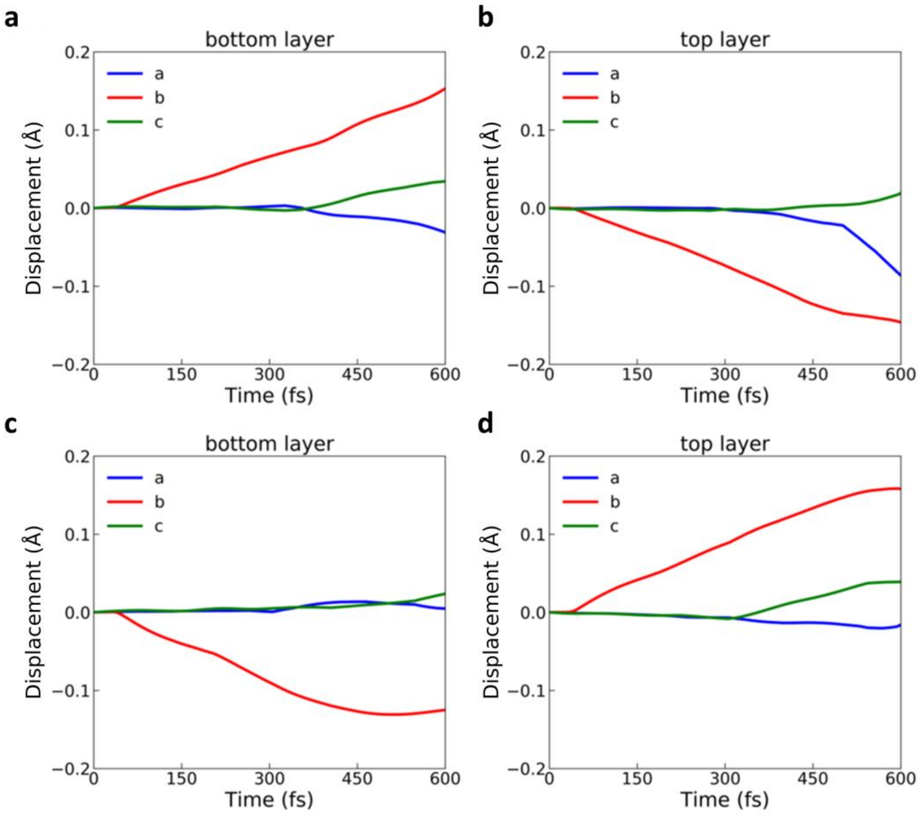


**Supplementary Fig. 5| Interlayer displacements along the *a*-axis and *c*-axis with the photon energy of 0.6 eV. a,b,** The averaged atomic displacement of one layer

along the *a*-axis (blue lines), *b*-axis (red lines) and *c*-axis (green lines) under LP-a excitations. **c,d,** are analogous to **a,b,** but under LP-b excitation.

**Supplementary Note 7: Non-centrosymmetric degree of $WTe_2$ during laser illumination**

Supplementary Fig. 6a and 6b show the lattice structure of two $WTe_2$ phases, i.e., non-centrosymmetric $T_d$ phase and centrosymmetric 1T′(*) phase. Both two phases have an orthorhombic unit cell and can transform each other via interlayer shear displacement[1]. To characterize the non-centrosymmetric degree (NCD) of $WTe_2$ under laser illumination, a structure factor *d* is introduced, which represents the structural difference between the two phases. The initial value of *d* is 0.405 Å, meaning that if the bottom-layer displacement with respect to the top-layer is 0.405 Å and the two layers move towards each other, the $T_d$ phase will transform to 1T′(*) phase. The decrease of *d* leading to the restoring of inversion symmetry, whereas the increase of *d* indicates that the non-centrosymmetric order is further enhanced. Supplementary Fig. 6d shows the evolution of *d* under the linearly polarized laser pulses. The decrease of *d* when the laser field is polarized at 45° off the crystallographic *a*-axis can be compared with the time-evolution of experimental detected SHG intensity (Fig. 4e in Ref. [1]). In the main text, the normalized *d* and SHG intensity are displayed in Fig. 5c. Under this polarization condition, the interlayer shear movements lead to the restoring of inversion symmetry (Supplementary Fig. 6c), which is consistent with the experimental observations. We notice that the atomic motions are slower than that under the LP-a and LP-b excitations ($\approx$ 0.076 Å/ps), which might be attributed to the fact that the accumulative effect of photoexcited carriers is cancelled out by its two polarization components.

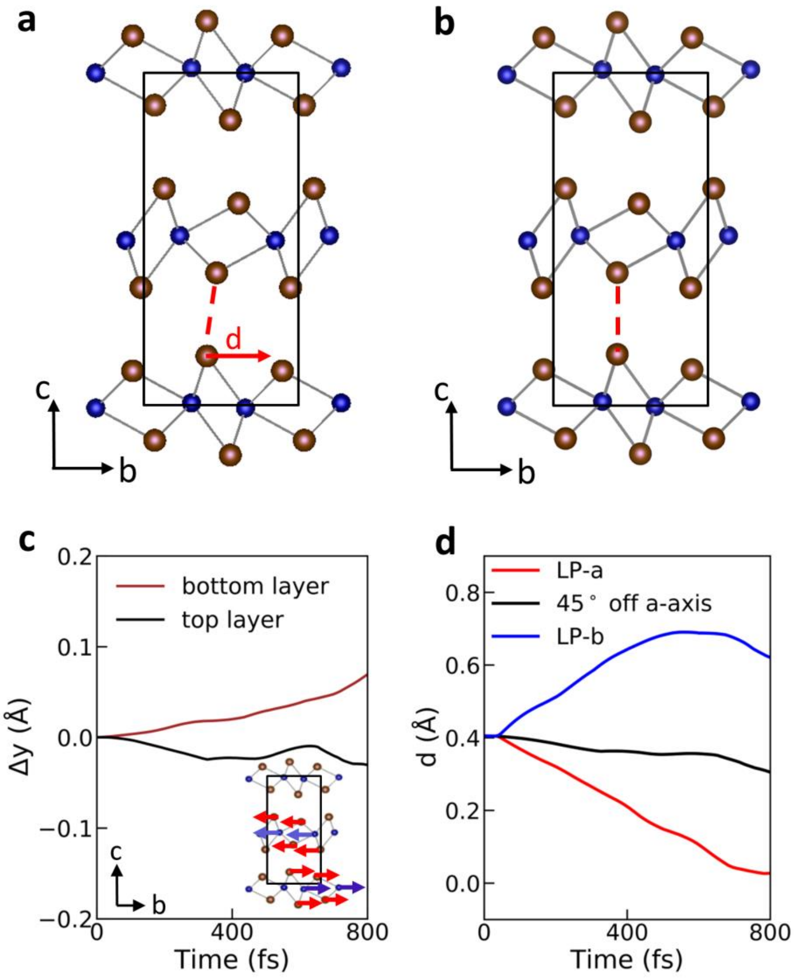


**Supplementary Fig. 6| Non-centrosymmetric degree of $WTe_2$ during laser illumination. a,b,** Lattice structure of $T_d$ (**a**) and 1T′(*) (**b**) phases of $WTe_2$, structure factor *d* is used to represent the interlayer difference between the two phases. **c**, Interlayer shear displacement when the pump fields are polarized at 45° off the crystallographic *a*-axis. The other laser parameters are same as those shown in Supplementary Fig. 1a. **d,** Time-dependent *d* under three excitation conditions.

**Supplementary Note 8: Electronic excitation and ionic movement with the photon energy of 1.5 eV**

Here, we show that when the carrier excitations are far away from the Weyl cone, the shear motion always starts from the $T_d$ phase to the centrosymmetric phase regardless of polarization, i.e., polarization isotropic. To demonstrate that, laser pulses with a photon energy of 1.5 eV are applied to $T_d$-$WTe_2$ (Supplementary Fig. 7a). Following the same analysis methods in the main text, we find that both electrons and holes are excited to the energy levels far away from $E_f$ no matter what polarization is adopted (Supplementary Fig. 7c and 7e). The resultant shear mode is polarization isotropic and towards restoring the inversion symmetry (Supplementary Fig. 7d and 7f), consistent with previous experimental observations[1,2].

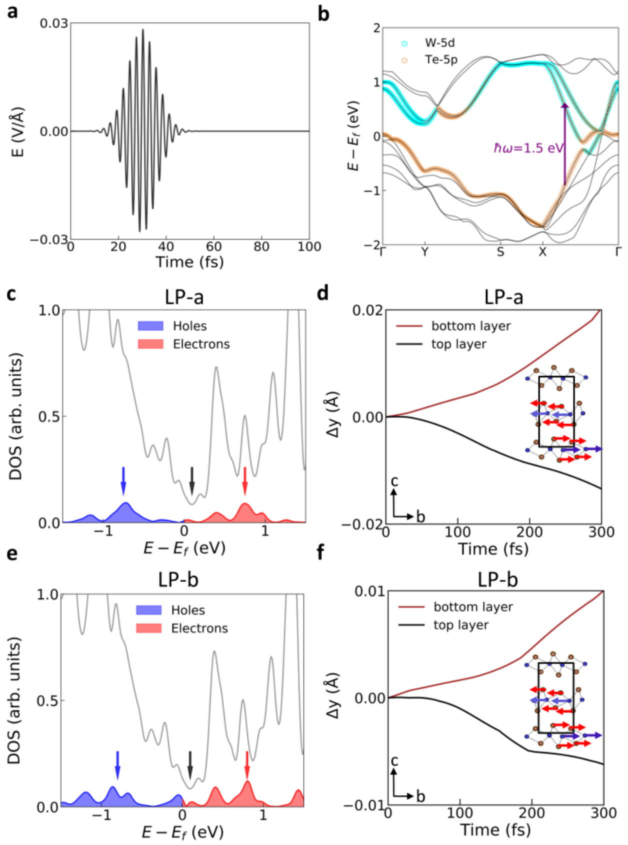


**Supplementary Fig. 7| Carrier excitation and ionic movement with the photon energy of 1.5 eV. a,** Applied laser pulse along the in-plane direction of $T_d$-$WTe_2$ with the laser strength $E_0 = 0.028$ V/Å and the photon energy is 1.5 eV. The pulse width is 6 fs and reaches it maximum at 30 fs. **b**, Schematic illustration of the carrier excitation under the light field. **c,** Energy distribution of the excited carriers with LP-a

excitation, where the red and blue arrows denote the peaks of the excited electrons and holes, which locate at the energy level of 0.8 eV and −0.7 eV, respectively. **d,** Shear displacements of two layers along *b*-axis. **e**, **f,** are analogous to **c**, **d,** but under LP-b excitation.

**Supplementary Note 9: Polarization-isotropy versus polarization-anisotropy for the interlayer shear displacement: the dependence on photon energy**

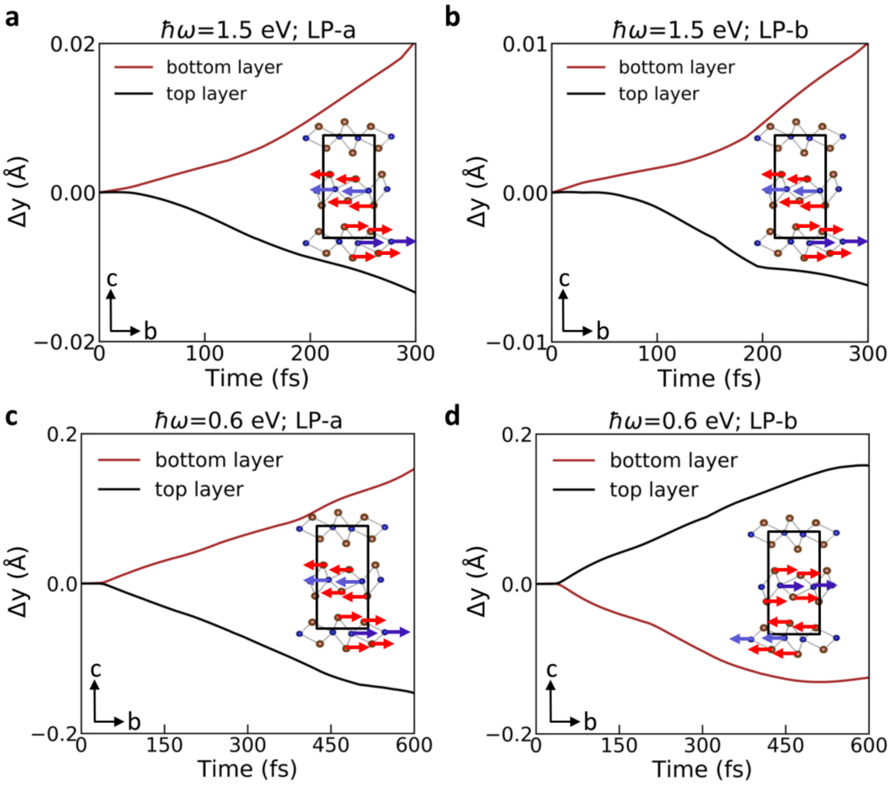


**Supplementary Fig. 8| Polarization dependence of interlayer shear displacements upon excitation with a different photon energy. a,b,** For a larger photon energy ($\hbar\omega$ = 1.5 eV), polarization-isotropic interlayer displacements are expected. **c,d** If the optical transitions are around the Weyl nodes ($\hbar\omega$ = 0.6 eV), the interlayer shear displacements are strongly polarization anisotropic.

**Supplementary Note 10: Electron correlation effects**

The LDA or GGA functionals tend to over-delocalize electrons and underestimate the correlation effects. The DFT/TDDFT+U approach is an effective method that might help to improve the accuracy of theoretical description of the strongly

correlated systems[3]. The correlation effect induced modification of band structure[4] and the ultrafast Lifshitz transition[5] are important knowledge in understanding the topological physics. However, the main points that addressed in this work is the topologically phase transition induced by the lattice dynamics, which can be controlled via orbital-selective photoexcitation. The above two mechanisms are not the same thing, but they are necessarily linked, and complementary. We believe that the real electronic dynamics is the combination of the two pictures.

To demonstrate that the correlation effect plays a minor role in our work, band structure of $T_d$-$WTe_2$ is calculated based on DFT+$U$ approach. Supplementary Fig. 9 shows the atomic-orbital projected band structure with $U$=0 (standard PBE calculation) and $U$=2 eV. In our work, the atomic orbitals around the Weyl node and their symmetry characteristics are of vital importance, which nearly identical with different Hubbard $U$. Based on that, we proposed that the correlation effect will not influence the main conclusions in this work, i.e., a switchable interlayer shear motion with respect to linear light polarization.

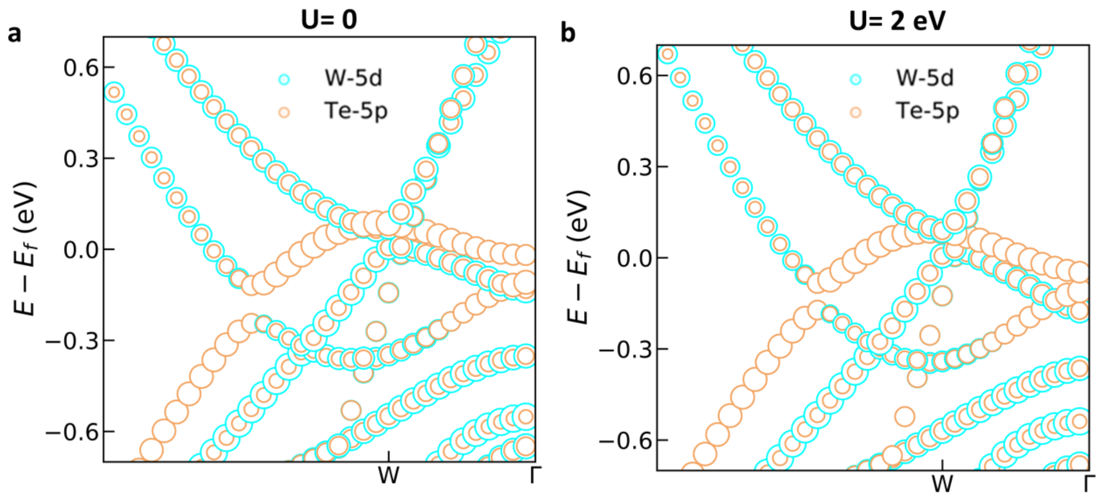


**Supplementary Fig. 9| PBE+*U* band structures of $T_d$-$WTe_2$ around the Weyl node.** **a,** $U$=0 and **b,** $U$=2 eV.

## Supplementary Note 11: Floquet driving effects

Based on the Floquet picture, the electronic band structure can also be modulated through the direct laser-electron interactions. For example, Hübener *et al*

demonstrated that Floquet-Weyl point trajectories can be controlled in $Na_3Bi$ via applying two time-delayed circularly polarized laser pulses[6]. In their work, circularly polarized lights are used to split the Dirac fermion into two Weyl fermions by introducing the time reversal symmetry breaking, and the tunable delay time leads to varied field strength. While in $T_d$-$WTe_2$, the robust Weyl fermions are already exist intrinsically due to the absence of inversion symmetry, linearly polarized laser will not change the Weyl point positions but only lead to some replicated bands.

Let us consider the model Hamiltonian[7]

$$H_0(\mathbf{k}) = Ck_x + \mathbf{k} \cdot \boldsymbol{\sigma}, \qquad (14)$$

which realize a low-energy effective Weyl point with a +1 Chern number. The parameter $C$ can adjust the type of this Weyl point and $\boldsymbol{\sigma} = (\sigma_x, \sigma_y, \sigma_z)$ is the Pauli matrix vector. Without loss of generality, we set the vector potential of laser as $\mathbf{A}(t) = A_0(cos\Omega t, 0, 0)$. The time-dependent Hamiltonian $H(\mathbf{k}, t)$ is introduced by Peierls substitution

$$H(\mathbf{k}, t) = H_0\big(\mathbf{k} - \mathbf{A}(t)\big)$$

$$= C(k_x - A_0 cos\Omega t) + (k_x - A_0 cos\Omega t)\sigma_x + k_y\sigma_y + k_z\sigma_z. \quad (15)$$

We construct the effective Floquet Hamiltonian perturbatively from the Fourier decomposition of $H(\mathbf{k}, t)$

$$H_0 = H_0(\mathbf{k}), \qquad (16)$$

$$H_{\pm 1} = -CA_0 - A_0\sigma_x, \qquad (17)$$

where $H_n = \frac{1}{T}\int_0^T H(\mathbf{k}, t)e^{it\Omega n}dt$. Since the commuter of $H_{-1}$ and $H_1$ equals to 0, the effective Floquet Hamiltonian is

$$H_{eff} = H_0 + \frac{[H_{-1}, H_1]}{\Omega} + \mathcal{O}(A_0^4) = H_0(\mathbf{k}) + \mathcal{O}(A_0^4), \qquad (18)$$

which retains the characteristics of the original band structure. The linear polarized light thus keeps the Weyl point positions unchanged. The only effect caused by direct laser-electron interaction might be the presence of Floquet band replicas due to the virtual processes of photon absorption and emission.

**Supplementary Note 12: Electronic dynamics due to photoexcitation**

Recently, Beaulieu *et al.* proposed that the dynamics of the wavefunctions and change of electronic structure due to photoexcitation played a key role in their experiment, even before lattice effects become important[5]. In the present work, the Weyl node separation (or annihilation) and the dynamics of the wavefunctions are indirectly linked via the atomic displacements. The direct impact of the electronic dynamics due to photoexcitation can be investigated by monitoring the time-evolution of electronic population in the momentum space with a fixed atomic geometry, as shown in Supplementary Fig. 10.

Supplementary Fig. 10a to 10c show carrier occupation difference between the ground ($n$(0 fs)) and excited ($n(t)$) states during the laser illumination. It is obvious that at the end of laser pulse, most of excited carriers are relaxed to energy region around the Weyl node. After the end of laser pulse (t = 70 fs), the intraband carrier scattering is the dominant interaction of the system (Supplementary Fig. 10d), which might plays an important role in influencing the carrier-phonon couplings and the topological phase transition. Therefore, the laser induced real-time evolution of the carrier-carrier and carrier-phonon couplings are intrinsically included in our approach, while we focus on the modulation effect of lattice dynamics in the present work.

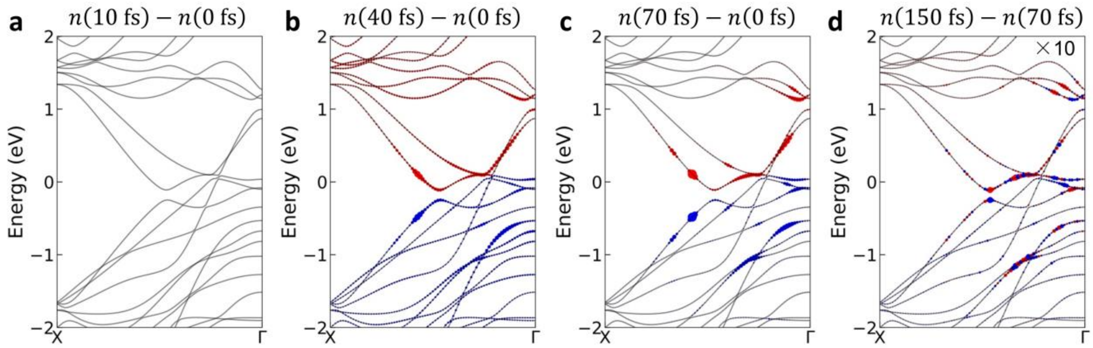


**Supplementary Fig. 10| Dynamics of carrier excitation in the momentum space with fixed atomic geometry.** Panels a to c show differences in carrier occupation between the ground ($n$(0 fs)) and excited ($n(t)$) states during laser illumination. Panel d describes carrier population changes from that at the end of laser pulse (70 fs) to that at 150 fs. The red (blue) dots represent the increase (decrease) of electronic occupation.

## Supplementary References


1. Sie, E. J., *et al.* An ultrafast symmetry switch in a Weyl semimetal. *Nature* 565, 61 (2019).
2. Hein, P., *et al.* Mode-resolved reciprocal space mapping of electron-phonon interaction in the Weyl semimetal candidate Td-$WTe_2$. *Nat Commun* 11, 2613 (2020).
3. Tancogne-Dejean, N., Oliveira, M. J. T. & Rubio, A. Self-consistent DFT+U method for real-space time-dependent density functional theory calculations. *Phys Rev B* 96, 245133 (2017).
4. Di Sante, D., *et al.* Three-dimensional electronic structure of the Type-II Weyl semimetal $WTe_2$. *Phys Rev Lett* 119, 026403 (2017).
5. Beaulieu, S., *et al.* Ultrafast Light-Induced Lifshitz Transition. Preprint at https://arxiv.org/abs/2003.04059 (2020).
6. Hubener, H., Sentef, M. A., De Giovannini, U., Kemper, A. F. & Rubio, A. Creating stable Floquet-Weyl semimetals by laser-driving of 3D Dirac materials. *Nat Commun* 8, 13940 (2017).
7. Soluyanov, A. A., *et al.* Type-II Weyl semimetals. *Nature* 527, 495-498 (2015).
